# A low frequency persistent reservoir of a genomic island in a pathogen population ensures island survival and improves pathogen fitness in a susceptible host

**DOI:** 10.1111/1462-2920.13482

**Published:** 2016-08-26

**Authors:** Helen C. Neale, Robert Laister, Joseph Payne, Gail Preston, Robert W. Jackson, Dawn L. Arnold

**Affiliations:** ^1^ Centre for Research in Bioscience, Faculty of Health and Applied Sciences The University of the West of England Frenchay Campus Bristol BS16 1QY UK; ^2^ Department of Engineering Design and Mathematics The University of the West of England Frenchay Campus Bristol BS16 1QY UK; ^3^ Department of Plant Sciences University of Oxford Oxford OX1 3RB UK; ^4^ School of Biological Sciences University of Reading Reading RG6 6UR UK

## Abstract

The co‐evolution of bacterial plant pathogens and their hosts is a complex and dynamic process. Host resistance imposes stress on invading pathogens that can lead to changes in the bacterial genome enabling the pathogen to escape host resistance. We have observed this phenomenon with the plant pathogen *Pseudomonas syringae* pv. *phaseolicola* where isolates that have lost the genomic island PPHGI‐1 carrying the effector gene *avrPphB* from its chromosome are infective against previously resistant plant hosts. However, we have never observed island extinction from the pathogen population within a host suggesting the island is maintained. Here, we present a mathematical model which predicts different possible fates for the island in the population; one outcome indicated that PPHGI‐1 would be maintained at low frequency in the population long term, if it confers a fitness benefit. We empirically tested this prediction and determined that PPHGI‐1 frequency in the bacterial population drops to a low but consistently detectable level during host resistance. Once PPHGI‐1‐carrying cells encounter a susceptible host, they rapidly increase in the population in a negative frequency‐dependent manner. Importantly, our data show that mobile genetic elements can persist within the bacterial population and increase in frequency under favourable conditions.

## Introduction

Bacterial genomes can evolve rapidly in different environmental conditions, typically through mechanisms such as horizontal gene transfer (HGT) and loss of mobile regions of DNA. Potential mobile regions of DNA include plasmids, transposons and genomic islands (GIs). GIs are regions of the genome that are present in some strains of bacteria but not others, are normally associated with specific integration sites in the genome such as tRNA loci and contain genes that may be responsible for recombination and mobility such as integrases and pili (Hacker and Kaper, 2000; Hacker and Carniel, 2001; van der Meer and Sentchilo, 2003). A number of GIs have been demonstrated to have roles in the virulence of their hosts, for example, the islands PAPI‐1 and PAPI‐2 contribute significantly to the virulence of *Pseudomonas aeruginosa* PA14 in acute pneumonia and bacteremia models (Harrison *et al*., 2010) and loss of island SPI‐4 from *Salmonella enterica* serovars Typhimurium and Enteritidis attenuates oral virulence in mice (Kiss *et al*., 2007). A number of plant pathogens also carry GIs (Arnold *et al*., 2003), for example, the *hrp/hrc* (hypersensitive response and pathogenicity/conserved) genes, which encode a type 3 secretion system used to deliver effector proteins, are carried on a GI with tripartite mosaic structure in *Pseudomonas syringae* (Alfano *et al*., 2000). Similarly, the virulence gene *hopAB1* (*virPphA*), which is essential for virulence of *P. syringae* pv. *phaseolicola* (*Pph*), is found on a GI carried by a 154 kb plasmid (Jackson *et al*., 1999), and in *Streptomyces acidiscabies*, the production of a phytotoxin called thaxtomin relies on genes carried on a 26 kb GI (Bukhalid *et al*., 2002). GIs also continue to be identified, for example, a collection of GIs have been recently identified in *P. syringae* pv. *actinidiae*, which causes the devastating disease of bacterial canker on kiwifruit (Butler *et al*., 2013; McCann *et al*., 2013).

*Pph* is a plant pathogen that causes halo blight disease of beans, the molecular genetics of which have been studied for many years, and, as such, it is often used as a model plant pathogen (Arnold *et al*., 2011). *Pph* has been subdivided into a number of races based on the gene‐for‐gene interaction between effector genes in the pathogen and resistance genes in the host (Taylor *et al*., 1996a,b). An example of this gene‐for‐gene interaction is between the effector gene *avrPphB* (also named *hopAR1)* carried by *Pph* race 4 strain 1302A, which matches resistance gene *R3* and induces a rapid resistance reaction called the hypersensitive response (HR) or effector triggered immunity (ETI) in bean cultivar Tendergreen (TG) (Jenner *et al*., 1991; Jones and Dangl, 2006). Induction of the HR leads to programmed cell death at the infection site, and results in the development of antimicrobial conditions due to the production of reactive oxygen and nitrogen species (Fones and Preston, 2012), and secondary metabolites such as phytoalexins (Mur *et al*., 2008). AvrPphB is a cysteine protease (Shao *et al*., 2002) that targets the protein serine/threonine kinase PBS1 in Arabidopsis, which in turn triggers cytoplasmic immune receptor RPS5‐specified ETI. However, AvrPphB also has a virulence function because in the absence of RPS5, it inhibits pathogen associated molecular pattern‐triggered immunity (PTI) by cleaving additional PBS1‐like kinases (Zhang *et al*., 2010).

Inoculation of 1302A in TG results in the evolution of a virulent strain derived from 1302A (named RJ3), due to the selection pressure on the pathogen to evade the HR triggered by *avrPphB*. *avrPphB* resides on a 106 kb GI designated PPHGI‐1 (Jackson *et al*., 2000; Pitman *et al*., 2005). The PPHGI‐1 integrase *xerC* gene enables the excision of PPHGI‐1 from the chromosome to form a circular molecule, leading to down‐regulation of *avrPphB* transcription (Godfrey *et al*., 2011). PPHGI‐1 also has the potential to self‐replicate (Lovell *et al*., 2011) and can be transferred between strains of *Pph* by *in planta* transformation (Lovell *et al*., 2009). During extended infection within the resistant plant, PPHGI‐1 is lost from the genome of *Pph* 1302A through natural selection, causing a change in host range and the production of water‐soaked lesions typical of disease. Comparison of the growth rates of bacteria with and without PPHGI‐1, inoculated at equal densities in the susceptible host, shows no advantage to carrying or losing the island (Pitman *et al*., 2005).

We have built upon this phenomenon to develop an experimental evolution system that allows us to follow the dynamics of pathogen evolution. This relies on passaging of bacteria within plants, for example to follow the loss of PPHGI‐1 from *Pph* strain 1302A in leaves of TG. In doing so, we have observed that PPHGI‐1 is rapidly lost from the bacterial population, with around 95% of the population having lost the island by week five (Pitman *et al*., 2005; Lovell *et al*., 2011). However, over a number of independent experiments we have never observed 100% PPHGI‐1 loss from the bacterial population.

We hypothesised that there is a threshold at which strains expressing AvrPphB either: (i) trigger plant cell death leading to island loss or (ii) are at a low enough frequency that the effects are suppressed by the dominant island‐less genotype, thus leading to island maintenance. We developed a mathematical model to determine the circumstances under which PPHGI‐1 would be maintained in the bacterial population long term; the model predicted that to be maintained the island must provide a growth advantage to the host bacterium, even when present at low frequencies in a mixed population. We then used an experimental approach to test the predictions resulting from the model. These data show that although host defence leads to significant island loss from the bacterial pathogen population, the island is able to persist until its bacterial host reaches a favourable environment upon which it rapidly increases in the bacterial population.

## Results

### PPHGI‐1 is lost from the genome of *Pph* 1302A::NCR during exposure to the HR

In our previous work, we have shown the loss of PPHGI‐1 through passaging of *Pph* 1302A in leaves of resistant bean cultivar TG. This was done by screening strains for the ability to cause HR and disease in bean pods: strains with the GI trigger the HR, those without the GI cause a water soaked lesion. Although this is a very sensitive assay, it is extremely time consuming, so to enable longer term studies we developed a rapid test for PPHGI‐1 loss using antibiotic resistance screening. *Pph* 1302A::NCR is a strain previously created that contains a kanamycin resistance cassette inserted into a predicted non‐coding region (NCR) of PPHGI‐1 (Pitman *et al*., 2005). To confirm that strain *Pph* 1302A::NCR behaved the same as wild‐type *Pph* 1302A with respect to PPHGI‐1 loss (and to also control for simple loss of the NCR insertion), we checked for PPHGI‐1 loss using our bean pod assay. We passaged *Pph* strain 1302A::NCR though the resistant bean cultivar TG six times and recorded island loss by both assaying on TG bean pods and monitoring the frequency of kanamycin‐resistant bacteria within populations. Both methods gave the same result, showing the highest loss of PPHGI‐1 observed as 98% after six weeks (Fig. S1). Thus, the results for 1302A and 1302A::NCR are congruent. This also correlated with our previous work, where we have never observed 100% loss of PPHGI‐1 over this time scale (Pitman *et al*., 2005; Lovell *et al*., 2011). Therefore, we concluded that monitoring the loss of kanamycin resistance from 1302A::NCR should allow us to do longer term passaging studies without reliance on bean pod assays.

### Mathematical predictions for the long‐term dynamics of PPHGI‐1 retention

**Figure 1:**
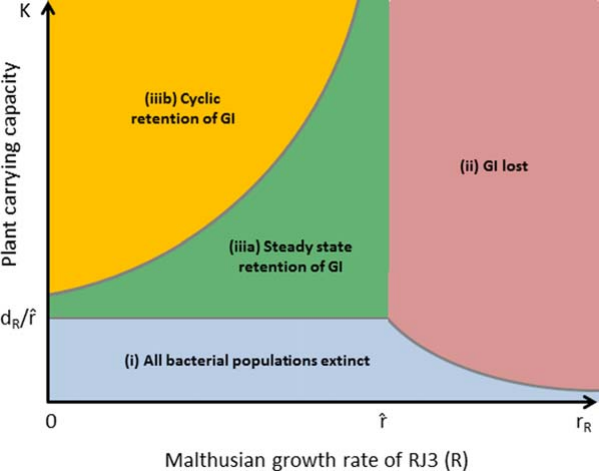
Schematic of the long‐term predictions of the mathematical model for all parameter values. In all cases, the plant survives. Parameter region (i) corresponds to eventual extinction of all bacterial populations, (ii) corresponds to survival of RJ3 only with 1302A becoming extinct, (iiia) corresponds to eventual co‐existence of RJ3 and 1302A at steady state (GI is retained) and (iiib) corresponds to cyclic (time‐periodic) coexistence of RJ3 and 1302A (GI is retained). The boundaries between regions (i), (ii) and (iiia) are given explicitly by $r_{R}=\hat{r}$, $K=1/\hat{r}$ and $r_{R}=d_{R}/K$, where $\hat{r}=r_{c}d_{R}/d_{c}$. The boundary curve between (iiia) and (iiib) is a locus of Hopf bifurcation points, marking the transition between steady state and cyclic GI retention.

Our passaging experiments indicated that a subpopulation of PPHGI‐1‐carrying cells was maintained in the population at a low frequency. We hypothesised that PPHGI‐1 conferred a fitness benefit to those cells during the suppression of antimicrobial conditions manifested by plant resistance, leading to island retention within the population. Firstly, to attempt to predict the effect of longer term exposure to a resistant plant environment on the ability of *Pph* 1302A to retain PPHGI‐1, we used a mathematical model (detailed in Supporting Information) to investigate the possible long term outcomes. Figure 1 summarises the key biological implications derived from our mathematical model. Essentially, the mathematical model predicts three qualitatively distinct long‐term (permanent) biological outcomes, depending on the relative values of the parameters in the model:

- *Bacterial extinction*. All bacterial populations (1302A and RJ3) die out, with the host plant surviving at its carrying capacity *K* (in the absence of bacteria the plant cell density would reach a steady‐state value of *K*) (region (i) in Fig. 1);
- *Loss of the GI*. The bacterial populations carrying the GI (1302A) die out but there is co‐existence of the GI‐free bacterial population (RJ3) with the host plant, the latter surviving at a level below its carrying capacity *K* (region (ii) in Fig. 1);
- *Retention of the GI*. There is co‐existence of bacterial populations both with the GI (1302A) and without the GI (RJ3), with the host plant surviving at a level below its carrying capacity *K* (regions (iiia) and (iiib) in Fig. 1).

It should be noted that these outcomes are stable in the sense that they do not depend on the particular initial population sizes (frequencies) of the bacteria or host and that small changes to the mathematical model would not affect the qualitative predictions (the system is ‘hyperbolic’).

The co‐existence predicted in (iii) can take one of two forms: (a) steady‐state or (b) cyclic. The term ‘steady‐state’ refers to population densities which are constant over time; the term ‘cyclic’ refers to population densities which are varying periodically over time. Note that (i)–(iii) are long‐term behaviours, i.e., population densities undergo transient dynamics before eventually settling down to one of these behaviours. It is outcome (iiia), which appears to correspond to our experimental results (Fig. S1). These three outcomes can be characterised in terms of just two of the model parameters, namely $r_{R}$, the intrinsic Malthusian growth rate of *R* (RJ3), and *K*, the carrying capacity of the host plant (see Supporting Information for the definition and role of these parameters). In particular, our mathematical model predicts that there exists a critical threshold value $\hat{r}$ such that the GI is retained if and only if $r_{R}$ is less than $\hat{r}$ and *K* is greater than $d_{R}/\hat{r}$ (see Fig. 1). The value $\hat{r}$ is given by $\hat{r}=r_{c}d_{R}/d_{c}$, where $r_{c}$ and $d_{c}$ are the intrinsic Malthusian growth and death rates of $B_{c}$ (wild‐type 1302A) respectively. We can write these conditions more elegantly and naturally by defining the two ratios, such as $\rho_{c}=r_{c\;}/d_{c}$ and $\rho_{R}=r_{R}/d_{R}$.

**Figure 2:**
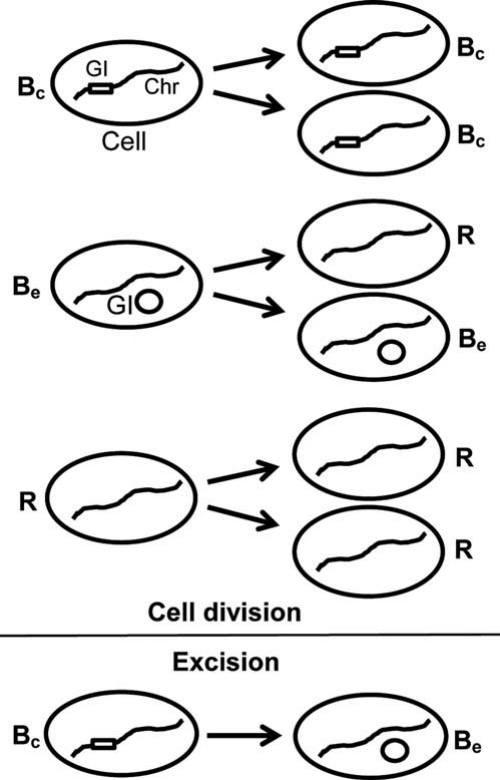
Dynamics of cell division of the three sub‐classes of bacterial cells and excision of the genomic island as defined for the mathematical model. GI, genomic island; Chr, chromosome; $B_{c}$, *Pph* 1302A GI in chromosome; $B_{e}$, Pph 1302A GI excised from chromosome; *R*, *Pph* RJ3 (GI lost).

**Figure 3:**
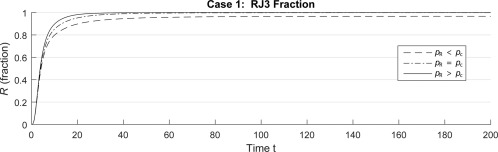
Mathematical predictions of the loss or retention of PPHGI‐1 during long‐term exposure of *Pseudomonas syringae* pv. *phaseolicola* strain 1302A to the plant's resistance response. *R* denotes 1302A after it has lost PPHGI‐1 (strain RJ3), $\rho_{c}=r_{c}/d_{c}$ and $\rho_{R}=r_{R}/d_{R}$ denote the reproductive ratios of RJ3 and 1302A ( $B_{c}$) respectively (where *r* and *d* are their respective natural growth and death rates). Case 1 corresponds to the case where $K>1/\rho_{c}$ (see Supporting Information). Time *t* = arbitrary units.

The quantity *r/d* is commonly referred to as the *reproductive ratio* of an organism having (Malthusian) growth rate *r* and natural death rate *d*, and represents the expected number of offspring produced during its natural lifespan *1/d*. The equivalent condition for GI retention is therefore that $\rho_{R}\;is\;less\;than\;\rho_{c}$ and *K* is greater than $1/\rho_{c}$, i.e., $\rho_{R}<\rho_{c}$ and $K>1/\rho_{c}$, providing a very simple algebraic criterion for GI retention and a simple biological interpretation in terms of the carrying capacity of the host plant and the reproductive ratios of the wild type bacteria $B_{c}$ (1302A) and the strain *R* (RJ3) (Fig. 2). The numerical simulations in Fig. 3 illustrate this, showing long term steady‐state GI retention for $\rho_{R}<\rho_{c}$ and GI loss for $\rho_{R}>\rho_{c}$ (and with *K* chosen such that $K>1/\rho_{c}$). The condition $K>1/\rho_{c}$ is equivalent to saying that wild type 1302A has an exponential *growth* phase (as opposed to exponential *decay*) when the host plant cell density is at its carrying capacity (*K*), in the absence of all other factors (such as host defence mechanisms and competition). This follows from the assumption that the natural growth rate response of 1302A is then $r_{c}K$ and its natural death rate is $d_{c}$, so exponential growth is possible only if $r_{c}K>d_{c}$, i.e., $K>1/\rho_{c}$.

### PPHGI‐1 is maintained in the population during long‐term exposure to the plant environment

**Figure 4:**
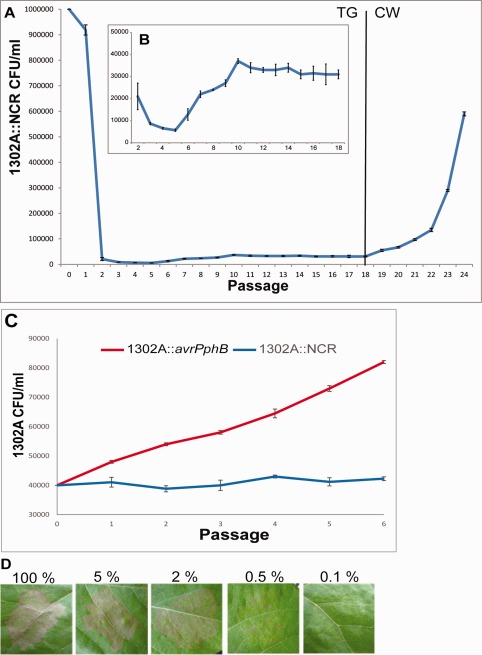
Population dynamics of *Pseudomonas syringae* pv. *phaseolicola* strain 1302A::NCR during exposure to host plants. A. Initially leaves of bean cultivar Tendergreen (TG) were inoculated at an inoculum density of OD600 0.1 with 2% *Pph* 1302A::NCR and 98% RJ3. After 7 days, the cells were harvested, diluted to the starting inoculum density, and reinoculated into a new leaf. This process was repeated 24 times, weeks 1–18 in TG followed by weeks 19–24 in bean cultivar Canadian Wonder (CW). (B) Zoomed in view between weeks 2 and 18. (C) The population of 1302A can increase *in planta* if *avrPphB* is inactivated. An *avrPphB* disruption mutant *Pph* 1302A::*avrPphB* and 1302A::NCR was passaged six times through resistant bean cv. TG at an initial inoculum concentration of 0.5% 1302A and 99.5% RJ3 to mimic that found at week 18. Colony‐forming units (CFUs) retaining kanamycin resistance and therefore PPHGI‐1 were counted after each passage. Mean is of three replicates ± SEM. (D) Phenotypes displayed by *Pph* 1302A::NCR on bean cv. TG at various concentrations of bacteria. Bean cv. TG leaves were inoculated with *Pph* 1302A::NCR OD600 0.1 concentrations of 100% (8 × 10^7^), 5% (4 × 10^6^), 2% (1.6 × 10^6^), 0.5% (4 × 10^5^) and 0.1% (8 × 10^4^) made up to 8 × 10^7^ cells ml^−1^ with *Pph* RJ3. These concentrations represent the levels of *Pph* 1302A::NCR observed at various time points in (A) and (B). Symptoms were recorded after 24 hours.

Based on the predictions made by the model described above a long‐term passaging experiment was carried out to investigate if PPHGI‐1 was maintained in the population over a longer time period than we had previously observed. We used strain *Pph* 1302A::NCR so that we could rapidly screen for the loss of PPHGI‐1 following plant exposure by screening for loss of kanamycin resistance. TG leaves were inoculated with a mixture of 2% 1302A::NCR and 98% RJ3 (this ratio mimics the population observed after six passages through TG starting with pure 1302A::NCR, Fig. S1). The experiment was carried out for a total of 18 weeks (Fig. 4A). We observed the rapid loss of PPHGI‐1 from the population and, therefore, a decrease in the proportion of the 1302A::NCR strain. Initially, this dropped to around 0.1% but then recovered to be stably maintained at 0.5% for the rest of the experiment. Figure 4B shows a zoomed in view of weeks 2–18 showing that the PPHGI‐1 containing strain drops to a very low level before invading from rare and being maintained in the population at a low level (approximately 0.5%) over time.

To confirm that the loss of PPHGI‐1 is being triggered by *avrPphB* induced HR we used a 1302A *avrPphB* insertion mutant (Lovell *et al*., 2011) and compared this to 1302A::NCR in a bean cv. TG passaging experiment (Fig. 4C); in each case the strains were mixed with RJ3 at a 0.5:99.5% ratio. When *avrPphB* is inactivated and therefore not triggering the HR, population numbers increase confirming that it is the HR activation by *avrPphB* that is causing the 1302A population to be held at a very low level.

PPHGI‐1 is thought to be lost from the genome of *Pph* 1302A during exposure to the plant's resistance mechanism because the antimicrobial environment generated by the HR favours PPHGI‐1 loss, enabling the evolved bacteria to avoid triggering the HR (Pitman *et al*., 2005; Arnold *et al*., 2007). Therefore, we considered at what concentration *Pph* 1302A was able to cause symptoms of the HR on bean leaves and whether there was a level at which 1302A could retain PPHGI‐1 without macroscopic symptoms of the HR being visible. TG leaves were inoculated with *Pph* 1302A::NCR OD_600_ 0.1 diluted to 100%, 5%, 2%, 0.5% and 0.1% with *Pph* RJ3 (Fig. 4D). This mimics the concentrations observed in the previous passaging experiments (Figs. 4A and S1). After 24 hours, the symptoms of the HR could be clearly seen in the leaves inoculated with 100%, 5% and 2% *Pph* 1302A. However, very weak HR symptoms could be seen at 0.5%, and no plant response was obvious when using a 0.1% density.

### Pph 1302A population frequency increases in a susceptible plant

As *Pph* 1302A excises PPHGI‐1 and loses the GI from its genome during the HR, it becomes identical to strain RJ3. However, even though *Pph* 1302A (B) could survive at a concentration low enough to significantly reduce HR symptoms, our model suggested that it would only be maintained in a population consisting mainly of RJ3 (R) if PPHGI‐1 conferred some fitness benefit. Therefore, to investigate this, the mixed bacterial population (99.5% RJ3 + 0.5% 1302A) harvested from the week 18 passage through TG leaves were inoculated into leaves of susceptible bean cultivar Canadian Wonder (CW) (Fig. 4A, weeks 19–24). Here, we observed that between weeks 19 and 24, the 1302A::NCR population increased 6.5‐fold, whereas the RJ3 population only increased 1.6‐fold (Fig S2). This increased population density suggests that 1302A has a fitness advantage over RJ3, when the HR is not a factor.

## Discussion

Our previous work has demonstrated that *Pph* strain 1302A carries a GI, PPHGI‐1, that harbours an effector gene, *avrPphB*, that is, responsible for the recognition of 1302A by a bean cultivar with the *R3* resistance gene (Jackson *et al*., 2000; Pitman *et al*., 2005). Yet although there is a strong selective pressure to lose PPHGI‐1 from the bacterial population during infection of this resistant bean cultivar, we have never observed 100% PPHGI‐1 loss from the population in any of our previous studies. This suggested three possibilities, that the excised GI integrates into the genome at a low frequency within the population to maintain a small population of island‐carrying bacteria, that the negative effects of the GI are suppressed at a critical minimal threshold and that the GI confers a fitness advantage to the minority. There are 100 predicted ORFs on PPHGI‐1 (Pitman *et al*., 2005), so it is possible that some of these may give the bacteria an advantage in certain environments. However, simply comparing the growth rates of the bacteria with and without PPHGI‐1 in a susceptible bean plant showed no clear fitness advantage to maintain PPHGI‐1 when comparing separate inoculations of 1302A and RJ3 (Pitman *et al*., 2005). We have also shown that there is no difference in growth rate if 1302A::NCR and RJ3 are co‐inoculated in a susceptible bean plant at equal densities (Fig. S3).

Here, we developed a mathematical model to try and predict the circumstances that would lead to the maintenance of PPHGI‐1 in a sub‐population of the bacteria. Our model suggested that PPHGI‐1 would be maintained indefinitely in a small proportion of the bacterial population if PPHGI‐1 gave the bacteria a fitness advantage once the selective pressure against the avirulence gene *avrPphB* was removed (specifically, if the intrinsic reproductive ratio of 1302A is greater than that of RJ3, with the assumption that the carrying capacity of the host plant is sufficiently large). We validated this prediction experimentally and went on to show that it is only when the strain containing PPHGI‐1 is at a very low level in the overall population (0.5%) that in cv. CW a fitness advantage can be seen, as the strain containing PPHGI‐1 can grow quicker than the island‐less strain and therefore increase as a fraction of the total population.

The mathematical model described here makes a number of assumptions, firstly, we have assumed that there remains an antimicrobial response at very low bacterial population densities, consistent with reports that a single bacterium is capable of eliciting the HR (Turner and Novacky, 1974). This is difficult to verify experimentally for such small population counts and their effects on the host plant may not even be visible macroscopically (Fig. 4D). It is conceivable that when PPHGI‐1 carrying strains are present at low frequencies in a population, antimicrobial activity may be insufficient to restrict bacterial growth. It would, therefore, be interesting to investigate models which include ‘threshold’ effects in which the antimicrobial response is negligible, which could facilitate the persistence of PPHGI‐1 even in the absence of a fitness benefit. Similarly, we have also assumed functional forms of mass‐action type in several parts of our model, including bacterial growth rates and the rate of island excision. It seems likely that more quantitatively accurate results could be obtained if growth rates due to nutrient uptake and antimicrobial inhibition were modelled via saturation functions of Monod type (i.e., there is an upper limit to the growth response). However, numerical simulations of such saturating effects (not reproduced here) show no important qualitative differences. We take the view that since not all parameter values are experimentally obtainable for this model, it is essentially qualitative in nature but is amenable to rigorous mathematical analysis. We are also aware that PPHGI‐1 is capable of HGT between bacteria cells (Lovell *et al*., 2009), which is not incorporated into the current model, which focuses on the maintenance of the island in the population and not in individual cells. However, we have also considered the effects of small rates of HGT in the mathematical model by carrying out a stability analysis entirely analogous to the one presented in the Supporting Information, which shows that the qualitative predictions, in particular those shown in Fig. 1, remain unchanged when the model includes HGT effects (data not shown).

There are two biologically interesting aspects of these results that warrant further consideration: (i) how is the PPHGI‐1 containing strain maintained at a low level in the population in the resistant host and (ii) what is the mechanism underlying its population increase in the susceptible host given that it does not appear to have an intrinsic growth advantage when populations are equal? For the maintenance of the GI at low level in the resistant host we have previously shown that PPHGI‐1 can excise and form a circular form outside of the chromosome and that when this occurs the avirulence gene *avrPphB* is down‐regulated (Godfrey *et al*., 2011). It is, therefore, possible that PPHGI‐1 strains carrying the excised GI escape the antimicrobial environment that seems to be needed to cause GI loss (Pitman *et al*., 2005) and that the excised island re‐integrates into the genome stochastically, maintaining a population of chromosomal GI carrying bacteria. It is also possible that at lower densities the PPHGI‐1 containing cells are benefiting from the suppression of plant defences by RJ3. In Young (1974) and Barrett *et al*. (2011), it was shown that the presence of virulent *P. syringae* strains significantly enhanced the growth of non‐pathogenic and non‐host strains in co‐inoculations and we may be observing a similar phenomenon here. However, our model suggests that PPHGI‐1 would only be maintained over an extended period of time if it conferred a fitness benefit and thus there may be as yet other undefined mechanisms of maintenance of PPHGI‐1. For example, it may be that *avrPphB* and other genes on PPHGI‐1 provide a fitness benefit to bacteria at lower densities, and it would be interesting to investigate the role of genes on PPHGI‐1 in the persistence of the island at low levels.

When the mixed population of bacteria (0.5% 1302A and 95.5% RJ3) is moved to a susceptible host 1302A grows more rapidly than RJ3, but the same effect was not observed when the two strains were inoculated at equal densities or *in vitro* (data not shown). This may be due to a fitness benefit conferred in the plant by the GI at low population density. This phenomenon of negative frequency‐dependent selection, where the fitness of a phenotype decreases as it becomes more common, is observed in other systems as a mechanism that maintains genotypes when they are rare in the population, thus favouring intraspecific diversity (Minter *et al.*, 2015). Possible mechanisms include a role for the GI in suppression of plant defences, which would provide a diminishing advantage as the proportion of plant cells in which defence responses have been suppressed increases, allowing bacteria that do not possess the GI to benefit from its activity. Here, we show that the population of bacteria is not genotypically homogeneous and a small proportion of cells still retain PPHGI‐1, which, when conditions change (e.g., interaction with susceptible plants) can have a fitness benefit, which is most apparent when bacteria are present at a low frequency.

Overall our results illustrate the maintenance of a mobile genetic element at low frequency within bacteria, helping to maintain a diversity of genetic material within their population. This enables the bacteria not only to now infect the previously resistant plant but also to rapidly colonise susceptible plants if the island‐containing genotype is dispersed to them. Given that these GI's are universally common amongst bacterial genera and include GI's in human pathogens containing virulence factors or antibiotic resistance genes, it is clear that this phenomenon can have serious implications for the persistence of genetic material. Moreover, the longevity of the GI as a persister population may make it difficult to displace the element from the bacterial population, thus causing difficulties in controlling disease outbreaks.

## Experimental procedures

### Bacterial and plant growth conditions

*Pph* 1302A::NCR (Pitman *et al*., 2005) and RJ3 (Jackson *et al*., 2000) were cultured at 25°C for 48 h on Kings B (KB) agar plates (Difco, UK). Overnight cultures were grown in Luria–Bertani media (Difco) at 25°C shaking at 200 rpm. Medium was supplemented with 25 mg ml^−1^ kanamycin where appropriate. *Phaseolus vulgaris* cultivar TG and cultivar CW were grown at 23°C, 80% humidity with a 16 h photoperiod. Pods were harvested from 8‐week‐old TG bean plants.

### The mathematical model

#### Mathematical modelling assumptions

We consider the population of bacterial cells to be composed of three distinct sub‐classes: (i) *Pph* 1302A cells with the GI located on their chromosomes ( $B_{c}$); (ii) *Pph* 1302A cells containing the excised form of the GI ( $B_{e}$) and (iii) RJ3 cells without the GI (*R*). We illustrate the relationship between these classes schematically in Fig. 2.

The presence of $B_{c}$ and $B_{e}$ cells, and hence of the effector gene *avrPphB* present on the GI, triggers an antimicrobial response (the HR) in the plant, via plant resistance gene *R3*. We denote the concentration of antimicrobial chemicals by (*A*) and assume that the antimicrobial response is proportional to the density of *Pph* 1302A cells and plant cells, in accordance with the law of mass action. The antimicrobial environment causes cell death in all three sub‐classes of bacterial cell as well as in the host plant itself and degrades at a constant rate. Simultaneously, the presence of the antimicrobial field induces excision of the GI from the chromosomes of $B_{c}$ cells, thereby converting these to the $B_{e}$ class (Pitman *et al*., 2005). We assume that the per capita rate of excision is proportional to the concentration of the antimicrobial field *A*.

We assume that the per capita rate of growth (replication) of the bacterial cells is proportional to the quantity of nutrients available and that the quantity of such nutrients is itself proportional to the density of living plant cells (*P*). For each of the three bacterial classes ( $B_{c},\;\;B_{e}$ and *R*), we consolidate these two constants of proportionality into a single parameter, namely the natural (Malthusian) growth rate ( $r_{c},\;\;r_{e}$ and $r_{R}$ respectively). In the absence of nutrients, the bacterial cells die at a constant per capita natural death rate ( $d_{c}$, $d_{e}$ and $d_{R}$). Finally, we assume that in the absence of bacteria, the plant cell density obeys a self‐limiting (logistic) growth law with carrying capacity *K* (i.e., in the absence of bacteria the plant cell density would reach a steady‐state value of *K*), but with a per capita death rate proportional to the bacterial cell densities in the presence of a bacterial population.

#### Mathematical formulation (see also Supporting Information)

There are four population densities representing the three sub‐classes of bacterial cells and one for the plant cells, which vary with time (assumed continuous), in addition to the concentration of the antimicrobial field:

$B_{c}(t)$ – population density at time t of bacterial cells having GI on chromosome;
$B_{e}(t)$ – population density at time t of bacterial cells having excised GI;
R(t) – population density at time t of bacterial cells without GI;
P(t) – population density at time t of plant cells;
A(t) ‐ concentration at time t of antimicrobial field.

We formulate our assumptions regarding the growth rates of these quantities as follows:

Growth rate of $B_{c}$ = (growth rate in presence of plant nutrients) – (natural death rate) – (death rate due to antimicrobials) – (rate of excision),
Growth rate of $B_{e}$ = (growth rate in presence of plant nutrients) – (natural death rate) – (death rate due to antimicrobials) – (rate of excision),
Growth rate of *R* = (growth rate in presence of plant nutrients) – (natural death rate) – (death rate due to antimicrobials),
Growth rate of *P* = (intrinsic logistic growth rate) – (death rate due to antimicrobials) – (death rate due to bacteria),
Rate of production of *A* = (HR response to bacteria) – (natural metabolic decay rate).

This growth rate model is most naturally and unambiguously cast as a system of five nonlinear ordinary differential equations. Full details of the mathematical model can be found in the Supporting Information together with its mathematical analysis and numerical simulations.

### *In planta* passaging

Overnight bacterial cell suspensions were washed, and the pellet resuspended in ¼ Ringers (Sigma, Aldrich, UK) followed by dilution to an optical density of 0.1 (OD_600_) before being infiltrated into bean leaves via a syringe and needle. After 7 days, inoculated tissue was harvested using a 1 cm core borer and each leaf disk was homogenized in 1 ml of ¼ strength Ringers solution (three independent replicates per time point). Bacteria were recovered by brief centrifugation to remove any remaining plant tissue and diluted to their original inoculated optical density (OD_600_), before being re‐infiltrated into fresh leaves. Recovered bacteria were also plated onto KB agar and KB agar plus Kanamycin (25 µg ml^−1^) at each passage. The resulting colonies were counted to determine the percentages of *Pph* 1302A::NCR and RJ3 present. For bean pod assays, TG pods were stab inoculated with recovered bacterial colonies and scored after 48h based on the inoculation site displaying watersoaking (disease) or browning and tissue collapse (HR). For phenotypic testing, *Pph* 1302A::NCR and RJ3 cell suspensions were mixed, so that they contained a 1302A::NCR proportion of 100%, 5%, 2%, 0.5% or 0.1% with a final combined OD_600_ of 0.1 (8 × 10^7^ cells ml^−1^) before being infiltrated into bean leaves as above. Symptoms were recorded and photographed after 24 h.

## Supporting information

Additional Supporting Information may be found in the online version of this article at the publisher's web‐site:

**Fig. S1.** PPHGI‐1 is lost from *Pseudomonas syringae* pv. *phaseolicola* 1302A::NCR during passaging though bean.
*Pph* 1302A::NCR was passaged six times (each passage 7 days) through resistant bean cv. TG. At each passage, 200 colonies were tested on TG pods (A) and via antibiotic selection (B) for the loss of PPHGI‐1 and the percentage loss recorded. Both tests showed the same result. Means are of three replicates ± SEM.
**Fig. S2.** *Pph* 1302A has a faster growth rate than RJ3 when the starting cell proportions are unequal.
The mixed bacterial population (99.5% RJ3 + 0.5% 1302A::NCR) harvested from week 18 passage (Fig. 4) through Tendergreen leaves was inoculated into leaves of susceptible bean cultivar Canadian Wonder and passaged six times. 1302A::NCR displays an increased growth rate, and its population increasing 6.5‐fold compared to 1.6‐fold for RJ3 between weeks 19 and 24. Mean is of three replicates ± SEM.
**Fig. S3.** *Pph* 1302A and RJ3 have similar growth rates *in planta* when the starting cell proportions are equal.
*Pph* 1302A::NCR and RJ3 were diluted to OD_600_ 0.1 and 250 µl of each strain mixed and inoculated into susceptible bean cultivar Canadian Wonder leaves. Samples were taken every 2 h and total colony forming units (CFU) calculated. Data shown are log_10_ CFU ml^−1^, and mean is of three replicates ± SEM.

Supporting Information
