# Supplementary material for: A low frequency persistent reservoir of a genomic island in a pathogen population ensures island survival and improves pathogen fitness in a susceptible host
Source: Environ Microbiol. 2016 Aug 26;18(11):4144–52. doi: 10.1111/1462-2920.13482 (PMC5573919; doi:10.1111/1462-2920.13482)
Supplement: Supplementary file 1 — Fig. S1. PPHGI‐1 is lost from Pseudomonas syringae pv. phaseolicola 1302A::NCR during passaging though bean. Pph 1302A::NCR was passaged six times (each passage 7 days) through resistant bean cv. TG. At each passage, 200 colonies were tested on TG pods (A) and via antibiotic selection (B) for the loss of PPHGI‐1 and the percentage loss recorded. Both tests showed the same result. Means are of three replicates ± SEM. Fig. S2. Pph 1302A has a faster growth rate than RJ3 when the starting cell proportions are unequal. The mixed bacterial population (99.5% RJ3 + 0.5% 1302A::NCR) harvested from week 18 passage (Fig. 4) through Tendergreen leaves was inoculated into leaves of susceptible bean cultivar Canadian Wonder and passaged six times. 1302A::NCR displays an increased growth rate, and its population increasing 6.5‐fold compared to 1.6‐fold for RJ3 between weeks 19 and 24. Mean is of three replicates ± SEM. Fig. S3. Pph 1302A and RJ3 have similar growth rates in planta when the starting cell proportions are equal. Pph 1302A::NCR and RJ3 were diluted to OD600 0.1 and 250 µl of each strain mixed and inoculated into susceptible bean cultivar Canadian Wonder leaves. Samples were taken every 2 h and total colony forming units (CFU) calculated. Data shown are log10 CFU ml−1, and mean is of three replicates ± SEM. [file EMI-18-4144-s001.docx]

**A**

**B**

**Figure S1. PPHGI-1 is lost from *Pseudomonas syringae* pv. *phaseolicola* 1302A::NCR during passaging though bean.** *Pph* 1302A::NCR was passaged six times (each passage 7 days) through resistant bean cv. TG. At each passage 200 colonies were tested on TG pods (A) and via antibiotic selection (B) for loss of PPHGI-1 and the percentage loss recorded. Means are of three replicates ±SEM.


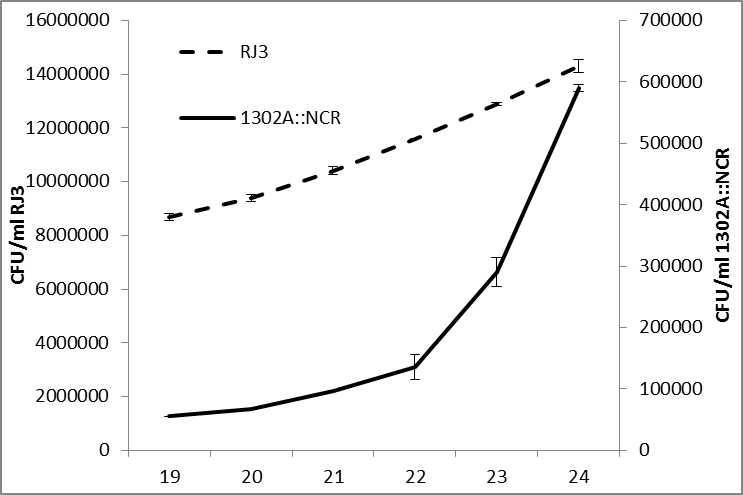


**Figure S2. *Pseudomonas syringae* pv. *phaseolicola* 1302A has a faster growth rate than RJ3 when the starting cell proportions are unequal.** The mixed bacterial population (99.5% RJ3 + 0.5% 1302A::NCR) harvested from the week 18 passage (Fig. 4) through Tendergreen leaves was inoculated into leaves of susceptible bean cultivar Canadian Wonder and passaged 6 times. 1302A::NCR displays an increased growth rate, its population increasing 6.5-fold compared to 1.6-fold for RJ3 between weeks 19-24. Means are of 3 replicates ±SEM.

**Figure S3. *Pseudomonas syringae* pv. *phaseolicola* 1302A and RJ3 have similar growth rates *in planta* when the starting cell proportions are equal.** *Pph* 1302A::NCR and RJ3 were diluted to OD_600_ 0.1 and 250µl of each strain mixed and inoculated into susceptible bean cultivar Canadian Wonder leaves. Samples were taken every 2 h and total colony forming units (CFU) calculated. Data shown is log_10_ CFU/ml and means are of 3 replicates ±SEM.
