# Supplementary material for: A low frequency persistent reservoir of a genomic island in a pathogen population ensures island survival and improves pathogen fitness in a susceptible host
Source: Environ Microbiol. 2016 Aug 26;18(11):4144–52. doi: 10.1111/1462-2920.13482 (PMC5573919; doi:10.1111/1462-2920.13482)
Supplement: Supplementary file 2 — Supporting Information [file EMI-18-4144-s002.pdf]

Mathematical Modelling: Analysis and Simulations

In these supplementary notes we provide a rigorous mathematical justification for some of the statements, results and diagrams presented in the main body of the paper. They consist essentially of a stability and bifurcation analysis of the steady-states of our mathematical model, together with some numerical computations and simulations.

1 The Mathematical Model

Our mathematical model comprises five ordinary differential equations (ODEs):

$$B_c'(t) = r_c P(t) B_c(t) - d_c B_c(t) - a_c A(t) B_c(t) - E A(t) B_c(t), \tag{1}$$
$$B_e'(t) = E A(t) B_c(t) - d_e B_e(t) - a_e A(t) B_e(t), \tag{2}$$
$$R'(t) = r_e P(t) B_e(t) + r_R P(t) R(t) - d_R R(t) - a_R A(t) R(t), \tag{3}$$
$$P'(t) = r_P P(t) \left(1 - \frac{P(t)}{K}\right) - P(t)\left(\delta_c B_c(t) + \delta_e B_e(t) + \delta_R R(t)\right) - a_P A(t) P(t), \tag{4}$$
$$A'(t) = \alpha P(t)(B_c(t) + B_e(t)) - d_A A(t). \tag{5}$$

This model was described less mathematically in the main body of the paper (see **Experimental Procedures**) via a set of growth rate equations. There, as in these supplementary notes,

- $B_c(t)$ denotes the population density at time t of bacterial cells having the genomic island (GI) on their chromosomes;
- $B_e(t)$ denotes the population density of bacterial cells which have excised the GI from their chromosomes but for which the GI still remains within the cell;
- $R(t)$ denotes the population density of bacterial cells which have lost the GI completely;
- $P(t)$ denotes the population density of host plant cells;
- $A(t)$ denotes the concentration of the antimicrobial field generated by the plant host's resistance mechanisms.

The parameters in (1-5) are all assumed to be positive and have the following biological interpretations (recall Figure 2 in the main document):

- r_c, r_e and r_R denote per capita growth rates of B_c, B_e and R respectively in the presence of nutrients (assumed proportional to P);

- d_c, d_e and d_R denote the per capita death rates of B_c, B_e and R respectively in the absence of nutrients;

- δ_c, δ_e and δ_R denote the per capita death rates of plant cells due to 'predation' by B_c, B_e and R respectively;

- a_c, a_e, a_R and a_P denote the per capita death rates of B_c, B_e, R and P respectively, caused by the toxic antimicrobial field A;

- K denotes the carrying capacity of the plant cells (i.e. the maximum sustainable density of plant cells in the absence of bacteria); r_P is the intrinsic (Malthusian) growth rate of the plant cells;

- E denotes the per capita excision rate of the GI in the presence of the antimicrobial field A;

- α is a rate constant for the production of the antimicrobial field due to the interaction of B_c and B_e with P (via the gene-for-gene interaction between *avrPphB* and $R3$); d_A denotes the (metabolised) degradation rate of the antimicrobial field A.

2 Existence and Stability of Steady-States

Let us first recall some basics of dynamical systems theory. Consider a system of n ODEs

$$x_i' = f_i(x_1, x_2, \ldots, x_n), \qquad i = 1, \ldots, n, \tag{6}$$

or in vector form

$$\mathbf{x}' = \mathbf{f}(\mathbf{x}). \tag{7}$$

We say that a point $\mathbf{x}^* = (x_1^*, x_2^*, \ldots, x_n^*)$ is a *steady-state* of (6) (equivalently (7)) if and only if $f_i(\mathbf{x}^*) = 0$ for all $i = 1, \ldots, n$ (equivalently $\mathbf{f}(\mathbf{x}^*) = \mathbf{0}$); i.e. $\mathbf{x}^*$ is a time-independent solution of (6) (equivalently (7)). The terms 'equilibrium point', 'fixed point', 'stationary point' and 'critical point' are also used synonymously in the mathematics literature but we prefer to use the term 'steady-state' here. A steady-state $\mathbf{x}^*$ is said to be *locally asymptotically stable* if the solution $\mathbf{x}(t)$ of (7) satisfies $\mathbf{x}(t) \to \mathbf{x}^*$ as $t \to \infty$ for all initial conditions $\mathbf{x}_0$ sufficiently close to $\mathbf{x}^*$; i.e. if the system is perturbed slightly from the steady-state at time $t = 0$ then it eventually settles back to that steady-state. In all that follows we will abuse terminology slightly by simply writing 'stable' instead of the more cumbersome (but technically correct) 'locally asymptotically stable'. We emphasize that stable states are of particular relevance since only the stable ones are physically (biologically) realizable.

There is a simple criterion to determine whether a given steady-state $\mathbf{x}^*$ is stable or unstable. Let $J(\mathbf{x}^*)$ denote the Jacobian of $\mathbf{f}$ at $\mathbf{x}^*$, i.e. $J(\mathbf{x}^*)$ is the $n \times n$ matrix with entries J_{ij} given by

$$J_{ij} = \frac{\partial f_i}{\partial x_j}(\mathbf{x}^*).$$

The eigenvalues of $J(\mathbf{x}^*)$ are then the roots of the characteristic equation

$$\det(J(\mathbf{x}^*) - \lambda I) = 0.$$

If all of these eigenvalues have negative real parts then $\mathbf{x}^*$ is stable. Conversely, if there exists at least one eigenvalue with positive real part then $\mathbf{x}^*$ is unstable.

In the notation of our model, $n = 5$, $(x_1, x_2, x_3, x_4, x_5) = (B_c, B_e, R, P, A)$ and the f_i $(1 \leq i \leq 5)$ are the right-hand sides of the five ODEs (1-5). One can verify easily that there are *at least* three steady-states of (1-5), namely

$$S_0: \quad \mathbf{x}_0^* = (0,0,0,0,0), \tag{8}$$

$$S_1: \quad \mathbf{x}_1^* = (0,0,0,K,0), \tag{9}$$

$$S_2: \quad \mathbf{x}_2^* = (0,0,R^*,P^*,0), \tag{10}$$

where

$$R^* = \frac{r_P}{\delta_R}\left(1 - \frac{d_R}{r_R K}\right), \qquad P^* = \frac{d_R}{r_R}. \tag{11}$$

S_0 is called the *trivial steady-state* (all components of $\mathbf{x}_0^*$ are zero), whilst S_1 and S_2 are called *semi-trivial steady-states* (some, but not all, components of $\mathbf{x}_1^*$ and $\mathbf{x}_2^*$ are zero). Biologically, S_1 corresponds to the situation where no bacteria are present (and hence no antimicrobial field) and the plant cell density is at its carrying capacity K. The steady-state S_2 is only biologically meaningful when $R^* > 0$, i.e. when $r_R/d_R > 1/K$. In such a case S_2 represents co-existence of plant cells (P^*) and RJ3 cells (R^*), and an absence of bacterial cells carrying the GI ($B_c = B_e = 0$), and consequently no antimicrobial field ($A = 0$) due to the absence of $R3$ genes. As we will see, there can exist other non-trivial steady-states in which both B_c, B_e and R all co-exist; this is particularly significant since our experimental data suggests that the GI is retained at a low (but non-zero) density *in planta*.

We now determine the stability of S_0, S_1, and S_2. Firstly, let us introduce the following notation:

$$\rho_c := r_c/d_c, \quad \rho_e := r_e/d_e, \quad \rho_R := r_R/d_R. \tag{12}$$

The quantities 'r/d' are commonly referred to as the 'reproductive ratio' of an organism and represents the expected number of 'offspring' produced via a Malthusian growth rate r during its natural

lifespan $1/d$ [1]. The Jacobian matrix of $\mathbf{f}$ is given by

$$J = \begin{pmatrix} J_{11} & 0 & 0 & r_c B_c & -B_c(a_c + E) \\ EA & J_{22} & 0 & 0 & EB_c - a_e B_e \\ 0 & r_e P & J_{33} & r_R R + r_e B_e & -a_R R \\ -\delta_c P & -\delta_e P & -\delta_R P & J_{44} & -a_P P \\ \alpha P & \alpha P & 0 & \alpha(B_c + B_e) & -d_A \end{pmatrix} \tag{13}$$

where

$$\begin{aligned} J_{11} &= r_c P - d_c - a_c A - EA, \\ J_{22} &= -d_e - a_e A, \\ J_{33} &= r_R P - d_R - a_R A, \\ J_{44} &= r_P\,(1 - 2P/K) - \delta_c B_c - \delta_e B_e - \delta_R R - a_P A. \end{aligned}$$

At S_0 (recall (8)) we obtain

$$J(\mathbf{x}_0^*) = \begin{pmatrix} -d_c & 0 & 0 & 0 & 0 \\ 0 & -d_e & 0 & 0 & 0 \\ 0 & 0 & -d_R & 0 & 0 \\ 0 & 0 & 0 & r_P & 0 \\ 0 & 0 & 0 & 0 & -d_A \end{pmatrix}$$

with eigenvalues given by

$$\lambda_1 = -d_c, \quad \lambda_2 = -d_e, \quad \lambda_3 = -d_R, \quad \lambda_4 = r_P, \quad \lambda_5 = -d_A.$$

Since $\lambda_4 > 0$ it follows that S_0 is always unstable. This is as expected, for in the absence of bacterial cells ($B_c = B_e = R = 0$), and hence of an antimicrobial field ($A = 0$), the plant cell density is assumed to satisfy the logistic equation

$$P' = r_P P\,(1 - P/K)\,,$$

for which $P = 0$ is unstable (and $P(t) \to K$ as $t \to \infty$).

At S_1 (recall (9)) we obtain

$$J(\mathbf{x}_1^*) = \begin{pmatrix} r_c K - d_c & 0 & 0 & 0 & 0 \\ 0 & -d_e & 0 & 0 & 0 \\ 0 & r_e K & r_R K - d_R & 0 & 0 \\ -\delta_c K & -\delta_e K & -\delta_R K & -r_P & -a_P K \\ \alpha P & \alpha P & 0 & 0 & -d_A \end{pmatrix}$$

with eigenvalues given by

$$\lambda_1 = r_c K - d_c, \quad \lambda_2 = -d_e, \quad \lambda_3 = r_R K - d_R, \quad \lambda_4 = -r_P, \quad \lambda_5 = -d_A. \tag{14}$$

Recalling (12) it follows that S_1 is stable if and only if

$$K < 1/\rho_c \quad \text{and} \quad \rho_R < 1/K. \tag{15}$$

Finally, consider S_2 (recall (10-11)) for $\rho_R > 1/K$, so that $R^* > 0$. We have that

$$J(\mathbf{x}_2^*) = \begin{pmatrix} r_c P^* - d_c & 0 & 0 & 0 & 0 \\ 0 & -d_e & 0 & 0 & 0 \\ 0 & r_e P^* & 0 & r_R R^* & -a_R R^* \\ -\delta_c P^* & -\delta_e P^* & -\delta_R P^* & r_P\left(1 - 2P^*/K\right) - \delta_R R^* & -a_P P^* \\ \alpha P^* & \alpha P^* & 0 & 0 & -d_A \end{pmatrix},$$

recalling (11). Three of the eigenvalues are given by

$$\lambda_1 = r_c P^* - d_c = d_c \left(\frac{\rho_c}{\rho_R} - 1\right), \quad \lambda_2 = -d_e, \quad \lambda_5 = -d_A, \tag{16}$$

with the remaining two (λ_3 and λ_4) being the roots of the quadratic equation

$$\lambda^2 + p\lambda + q = 0, \tag{17}$$

where

$$p = \delta_R R^* - r_P\left(1 - 2P^*/K\right) = r_P/\rho_R > 0 \quad \text{and} \quad q = r_R \delta_R P^* R^* > 0. \tag{18}$$

Since $p, q > 0$ the Routh-Hurwitz Criterion ensures that λ_3 and λ_4 have negative real parts. Hence we see that S_2 is both positive and stable if and only if

$$\rho_R > \max\left\{\frac{1}{K}, \rho_c\right\}. \tag{19}$$

It can be seen from this simple linear analysis that the stability of S_1 and S_2 depends only upon the bacterial reproductive ratios ρ_c and ρ_R (of B_c and R respectively) and the plant host's carrying capacity K. In what follows we will consider all other parameters as fixed (but arbitrary) and allow ρ_R to vary. For mathematical convenience (to ensure the steady-states are hyperbolic) we will assume that

$$K \neq 1/\rho_c. \tag{20}$$

This is an entirely reasonable assumption biologically, since the growth rate r_c and death rate d_c of B_c are defined independently from the carrying capacity K of the plant, hence it is highly

improbable that $r_c/d_c = 1/K$ by chance. Natural, stochastic fluctuations would also prevent equality from occurring in any real biological environment.

In order to fully describe the stability properties of S_1 and S_2 using (15) and (19) we must consider the two possible cases $K < 1/\rho_c$ and $K > 1/\rho_c$ separately. We summarize the two cases below. Recall also that S_0 is *always* unstable and that S_2 is only biologically relevant (non-negative) when $\rho_R > 1/K$.

Case 1: $K > 1/\rho_c$. S_1 is unstable for all $\rho_R > 0$; S_2 is unstable (and positive) for $1/K < \rho_R < \rho_c$ and stable (and positive) for all $\rho_R > \rho_c$.

Case 2: $K < 1/\rho_c$. S_1 is stable for $0 < \rho_R < 1/K$ and unstable for $\rho_R > 1/K$; S_2 is stable (and positive) for all $\rho_R > 1/K$.

For fixed ρ_c and K it is clear that there are at least two critical values of ρ_R (namely $\rho_R = 1/K$ and $\rho_R = \rho_c$) where bifurcations may occur at a simple zero eigenvalue, from which new families of steady-states may emerge.

3 Bifurcation of steady-states

We now fix ρ_c and K (and all other parameter values) and allow r_R to vary, i.e. we take r_R as bifurcation parameter. Note that since $\rho_R = r_R/d_R$, this is equivalent to varying ρ_R for d_R fixed, modulo rescaling. Despite Cases 1-2 being written in terms of ρ_R, we choose to take r_R as bifurcation parameter in order to avoid having to rescale (1-5), which we think would hinder the exposition below. To emphasize the dependence of (1-5) upon r_R we henceforth write $\mathbf{f}(\mathbf{x}, r_R)$ instead of $\mathbf{f}(\mathbf{x})$ in the ODE (7).

We introduce the set Σ of steady-states of (7) for any given r_R, defined by

$$\Sigma = \left\{(r_R, \mathbf{x}) \in \mathbb{R} \times \mathbb{R}^5 \mid \mathbf{f}(\mathbf{x}, r_R) = \mathbf{0}\right\}. \tag{21}$$

Clearly, Σ contains the following three branches corresponding to the three steady-states S_0, S_1 and S_2 found earlier:

$$\Sigma_0 = \left\{(r_R, \mathbf{x}) \in \mathbb{R} \times \mathbb{R}^5 \mid \mathbf{x} = \mathbf{x}_0^*\right\}, \tag{22}$$
$$\Sigma_1 = \left\{(r_R, \mathbf{x}) \in \mathbb{R} \times \mathbb{R}^5 \mid \mathbf{x} = \mathbf{x}_1^*\right\}, \tag{23}$$
$$\Sigma_2 = \left\{(r_R, \mathbf{x}) \in \mathbb{R} \times \mathbb{R}^5 \mid \mathbf{x} = \mathbf{x}_2^*\right\}. \tag{24}$$

(recalling (8-11)). Note that $\mathbf{x}_0^*$ and $\mathbf{x}_1^*$ do not depend on r_R, but $\mathbf{x}_2^*$ does. We also observe that $R^* = 0$ and $P^* = K$ when $r_R/d_R = 1/K$, so that the branches Σ_1 and Σ_2 intersect where $r_R = d_R/K$ and $\mathbf{x}_1^* = \mathbf{x}_2^*$. This is a consequence of the bifurcation of S_1 at a simple zero eigenvalue,

namely that of λ_3 in (14). Equivalently, one may view this as a bifurcation of S_2 at a simple zero eigenvalue, recalling (17-18) and the fact that $R^* = 0$. For reasons that will become clear below, we take the latter view and consider Σ_1 to bifurcate from Σ_2 and label this bifurcation point as Z_{21} (‘Z’ for ‘zero’ eigenvalue with the second digit in the subscript denoting the branch number in Σ which bifurcates *from* Σ_2), i.e.

$$Z_{21} = (r_R, \mathbf{x}) = (d_R/K, 0, 0, 0, K, 0). \tag{25}$$

The branch Σ_2 also undergoes a further (transverse) zero eigenvalue bifurcation at $r_R = d_R\rho_c$ (where $\lambda_1 = 0$ and $d\lambda_1/dr_R \neq 0$). We now perform a weakly nonlinear analysis at this bifurcation point in order to better understand the geometry and stability of the emerging bifurcating branches of new steady-states.

Let us consider the bifurcation occurring on branch Σ_2 at $r_R = d_R\rho_c$, considered only for $K > 1/\rho_c$, whereby $R^* > 0$. We denote this bifurcation point in Σ_2 by Z_{23}, i.e.

$$Z_{23} = (r_R, \mathbf{x}) = \left(d_R\rho_c, 0, 0, \frac{r_P}{\delta_R}\left(1 - \frac{1}{\rho_c K}\right), \frac{1}{\rho_c}, 0\right). \tag{26}$$

By classical bifurcation theory (see e.g. [2, 3, 5, 6]) there exists another branch of steady-states, Σ_3, intersecting Σ_2 transversally at Z_{23}. This branch can be parameterized (analytically, since $\mathbf{f}$ is analytic) as

$$r_R = d_R\rho_c + \gamma_4\varepsilon + O(\varepsilon^2), \quad \mathbf{x} = (0, 0, R^*, P^*, 0) + \varepsilon\mathbf{z} + O(\varepsilon^2) \tag{27}$$

where $\mathbf{z}$ spans the one-dimensional null space of the Jacobian $J(\mathbf{x}_2^*)$ when $r_R = d_R\rho_c$ and γ_4 is as yet undetermined. One easily shows that $\mathbf{z}$ is of the form

$$\mathbf{z} = (d_A, 0, z_3, z_4, \alpha/\rho_c), \tag{28}$$

so that in component form we have the following local parameterization of Σ_3:

$$\begin{aligned}
r_R(\varepsilon) &= d_R\rho_c + \gamma_4\varepsilon + O(\varepsilon^2), \\
B_c(\varepsilon) &= d_A\varepsilon + O(\varepsilon^2), \\
B_e(\varepsilon) &= 0 + O(\varepsilon^2), \\
R(\varepsilon) &= R^* + z_3\varepsilon + O(\varepsilon^2), \\
P(\varepsilon) &= P^* + z_4\varepsilon + O(\varepsilon^2), \\
A(\varepsilon) &= (\alpha/\rho_c)\varepsilon + O(\varepsilon^2).
\end{aligned}$$

We see that B_c and A are both positive for $\varepsilon > 0$ sufficiently small (and R and P are positive since $R^* > 0$ and $P^* > 0$ at $r_R = d_R\rho_c$). It is easy to show that $B_e(\varepsilon) \not\equiv 0$ on Σ_3 since the only steady-state solutions with $B_e = 0$ necessarily satisfy $B_c = A = 0$, yielding only S_0, S_1 or S_2.

We also observe that $B_c = O(\varepsilon)$ and $B_e = O(\varepsilon^2)$, so that B_e is at least an order of magnitude smaller than B_c on the scale of ε. We may now define a 'positive' part Σ_3^+ and a 'negative part' Σ_3^- corresponding to $\varepsilon > 0$ and $\varepsilon < 0$ respectively. We are not assured that $B_e > 0$ for $\varepsilon > 0$ via this linear theory alone but numerical simulations suggest that this is true, at least for the parameter values considered here.

4 Numerical simulations

Throughout this section the numerical values of the model parameters are taken as follows:

$$\begin{aligned} d_c &= d_e = d_R = 1,\ d_A = 0.4,\ a_c = a_e = a_R = 10,\ r_P = 10.2,\ r_c = 2,\ r_e = 3.6, \\ \delta_c &= 10.1,\ \delta_e = 10,\ \delta_R = 10.6,\ E = 1,\ a_P = 7,\ \alpha = 10. \end{aligned}$$

These values have been chosen arbitrarily. We have also run simulations for other choices of parameter values and whilst we have observed that the underlying bifurcation structure can behave in a slightly different quantitative manner, the qualitative behaviour remains unchanged and the implications for the biological model are the same.

We consider the Cases 1-2 separately and take r_R as the primary bifurcation parameter. We will see that it is also necessary to consider K as a secondary bifurcation parameter. The bifurcation diagrams were generated using the numerical continuation software package AUTO-07P, freely available via the internet [4]. Time plots of the solutions of the ODEs were generated using the numerical ODE solver *ode45* in MATLAB.

4.1 Case 1: $K > 1/\rho_c$

Recall that S_1 (plant cells only, no bacteria) is unstable for all $r_R > 0$ whilst S_2 (plant and RJ3 bacterial cells only) is non-negative and stable if and only if $r_R > d_R\rho_c$. As r_R increases from zero the steady-state S_2 (or more accurately, the corresponding branch Σ_2) undergoes two steady-state bifurcations: firstly at $r_R = d_R/K$ and secondly at $r_R = d_R\rho_c$ (these points being denoted by Z_{21} and Z_{23} respectively), resulting in the branches Σ_1 (corresponding to S_1) and $\Sigma_3^\pm$. Since S_2 is unstable for all $r_R < d_R\rho_c$, it follows (from Crandall-Rabinowitz [3]) that the branch Σ_1 is also unstable, at least locally near Z_{21}. Again by [3], and recalling (27), if $\gamma_4 > 0$ then Σ_3 is locally stable for $\varepsilon < 0$ (for then $r_R(\varepsilon) < d_R\rho_c$) and locally unstable for $\varepsilon > 0$ (for then $r_R(\varepsilon) > d_R\rho_c$). That is, if $\gamma_4 > 0$ then Σ_3^- is locally stable and Σ_3^+ is locally unstable. Conversely, if $\gamma_4 < 0$ then Σ_3^+ is locally stable and Σ_3^- is locally unstable. Of course, within Σ_3 it is only Σ_3^+ that may contain biologically meaningful (non-negative) steady-states.

4.1.1 ‘Intermediate’ Plant Carrying Capacity K

We begin by taking $K = 1$ (note that $K > 1/\rho_c = 1/2$). Figure S4 shows the bifurcation diagrams thus obtained in AUTO-07P. **As is standard in bifurcation theory, stable branches are denoted by solid lines and unstable ones by dashed lines.** The top left plot shows the location of the two bifurcation points Z_{21} and Z_{23} (labelled ‘1’ and ‘3’ respectively) together with the R-components of the associated bifurcating branches Σ_1 (labelled ‘2’), Σ_2^+ (labelled ‘4’) and $\Sigma_3^\pm$ (labelled ‘5’ and ‘6’). We include this figure to illustrate the complete structure of the bifurcating branches. The other five plots show only the non-negative (biologically relevant) components of the bifurcating branches, Σ_i^+.

We see in Figure S4 that Σ_2^+ is the only stable branch for $r_R > d_R\rho_c$ whilst Σ_3^+ is the only stable branch for $r_R < d_R\rho_c$. We also observe in the middle-right plot that $B_e > 0$ along Σ_3^+ (i.e. $\gamma_4 < 0$, recalling (27)).

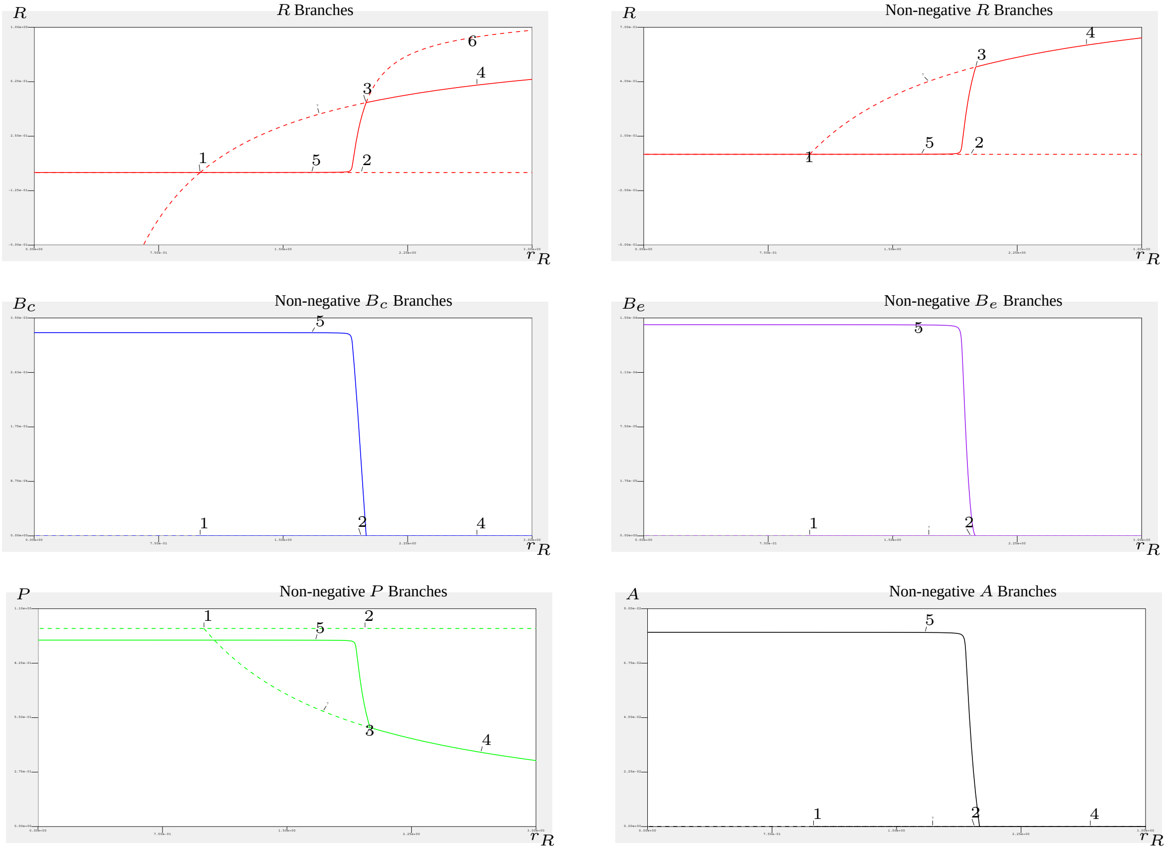

Figure S4: Bifurcation diagrams for Case 1 with $K = 1$. Diagram labels: (1) Z_{21}; (2) Σ_1 ; (3) Z_{23} ; (4) Σ_2^+; (5) Σ_3^+; (6) Σ_3^-.

Let us, for $r_R < d_R\rho_c$, denote by $\mathbf{x}_3^*$ the non-negative steady-state on the branch Σ_3^+ (branch

label ‘5’). We can then characterize the number and stability of non-negative steady-states of our model as follows:

- For $r_R > d_R\rho_c$ there are three non-negative steady-states $\{\mathbf{x}_0^*, \mathbf{x}_1^*, \mathbf{x}_2^*\}$. Only $\mathbf{x}_2^*$ is stable.
- For $r_R \in (0, d_R\rho_c)$ there are four non-negative steady-states $\{\mathbf{x}_0^*, \mathbf{x}_1^*, \mathbf{x}_2^*, \mathbf{x}_3^*\}$. Only $\mathbf{x}_3^*$ is stable.

One observes that as r_R decreases the number of non-negative steady-states increases. However, since only $\mathbf{x}_2^*$ and $\mathbf{x}_3^*$ may be stable in this case, we anticipate the following simple characterization of the long-term behaviour (recalling that $\rho_R = r_R/d_R$):

- if $\rho_R > \rho_c$ then the GI is not retained (B_c and B_e die out and R persists);
- if $\rho_R < \rho_c$ then the GI is retained (B_c, B_e and R all co-exist).

For these parameter values the mathematical model predicts retention of the GI if and only if the reproductive ratio of RJ3 (ρ_R) is smaller than the reproductive ratio of 1302A (ρ_c).

Figure S5 shows some numerically simulated time plots of the ODE model (1-5) when $K = 1$ in the three cases $\rho_R > \rho_c$, $\rho_R = \rho_c$ and $\rho_R < \rho_c$ (using ode45 in MATLAB). As expected we see (exponential) convergence to the stable steady-state $\mathbf{x}_2^*$ when $\rho_R > \rho_c$ and to $\mathbf{x}_3^*$ when $\rho_R < \rho_c$. We also see convergence to the steady-state $\mathbf{x}_2^*$ when $\rho_R = \rho_c$, but convergence is now slower (algebraic) due to the presence of a zero eigenvalue (and a centre manifold). The bottom right-hand figure shows the fraction of RJ3 cells of the entire bacterial population density over time, namely

$$\text{RJ3 Fraction} = \frac{R(t)}{B_c(t) + B_e(t) + R(t)}.$$

These qualitative mathematical results are in agreement with the experimental data shown in Figures 4a-b of the main document. The bottom right panel of Figure S5 appears as Figure 3 in the main document.

4.1.2 ‘Large’ Plant Carrying Capacity K

We now consider the same parameter values as above but for larger K, i.e. for $K = 15$. Figure S6 shows similar features to the $K = 1$ case (Figure S4), but now with two additional branches $\Pi^\pm$ (labelled ‘10’ and ‘11’) consisting of time-periodic (‘cyclic’) solutions $\mathbf{c}^\pm(t)$ which arise via secondary Hopf bifurcations from the branches $\Sigma_3^\pm$ (see top-left plot). We denote these secondary Hopf bifurcation points by $H^\pm$ (labelled ‘8’ and ‘9’) and the corresponding values of r_R by and $r^\pm$

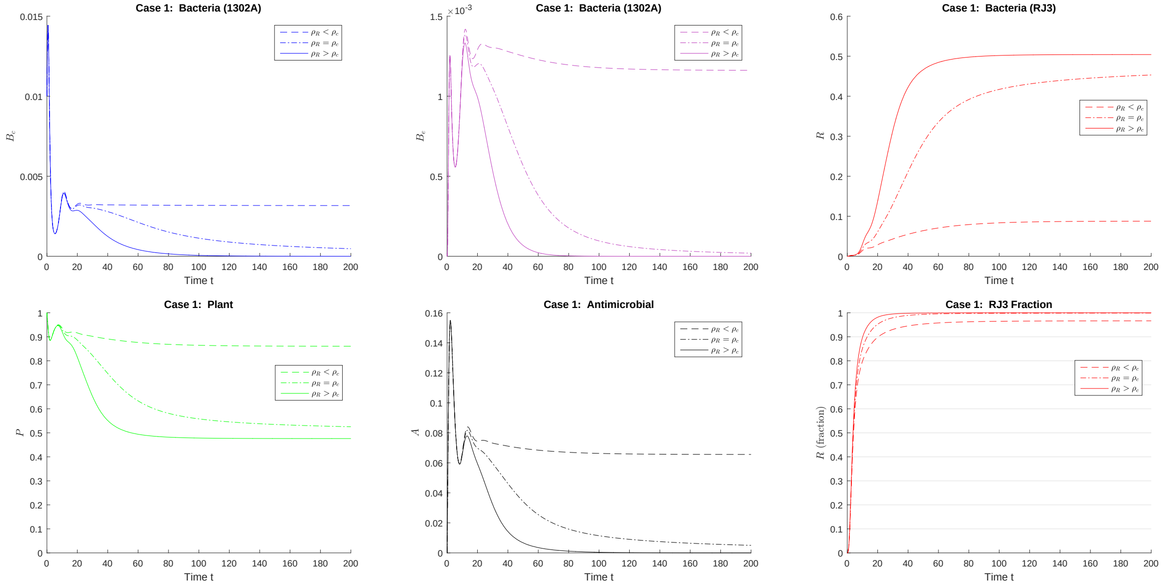

Figure S5: $K = 1$, $r_c = 2$, $r_e = 3.6$. Time plots of the ODE when: $r_R = 1.9$ ($\rho_R < \rho_c$), $r_R = 2.1$ ($\rho_R > \rho_c$) and $r_R = 2$ ($\rho_R = \rho_c$).

respectively. The branch Π^- comprises of periodic solutions which have at least one negative component and are thus irrelevant biologically. Conversely, the branche Π^+ is comprised of periodic solutions all of whose components are non-negative and therefore biologically relevant. These, and the other non-negative branches of steady-states, can be seen more clearly in the remaining five plots of Figure S6.

We summarize the bifurcation behaviour of non-negative solutions as r_R decreases thus:

- for $r_R > d_R\rho_c$ there are three non-negative steady-states $\{\mathbf{x}_0^*, \mathbf{x}_1^*, \mathbf{x}_2^*\}$. Only $\mathbf{x}_2^*$ is stable;
- for $r_R \in (r^+, d_R\rho_c)$ there are four non-negative steady-states $\{\mathbf{x}_0^*, \mathbf{x}_1^*, \mathbf{x}_2^*, \mathbf{x}_3^*\}$. Only $\mathbf{x}_3^*$ is stable;
- for $r_R \in (0, r^+)$ there are four non-negative steady-states $\{\mathbf{x}_0^*, \mathbf{x}_1^*, \mathbf{x}_2^*, \mathbf{x}_3^*\}$ and one non-negative limit cycle $\mathbf{c}^+$. Only $\mathbf{c}^+$ is stable.

Again as r_R decreases the number of non-negative steady-states increases, but this time with the appearance of a limit cycle. From stability considerations one therefore expects to see long-time convergence to $\mathbf{x}_2^*$ when $r_R > d_R\rho_c$, convergence to $\mathbf{x}_3^*$ when $r_R \in (r^+, d_R\rho_c)$ and convergence to $\mathbf{c}^+$ when $r_R < r^+$. Numerical simulations of the ODE model (1-5) in the latter case ($r_R < r^+$) are shown in Figure S7.

Once again, for the parameter values chosen here, the model predicts retention of the GI if and only if the reproductive ratio (ρ_R) of RJ3 is smaller than the reproductive ratio (ρ_c) of 1302A. However, for $K = 15$, it is also possible for the GI to be retained dynamically (cyclically) rather than at steady-state.

4.1.3 2-Parameter Bifurcation Loci

Evidently K plays some role in the qualitative structure of the bifurcation diagram since (for the parameter values chosen here) Hopf bifurcation of non-negative solutions occur when $K = 15$ but not when $K = 1$. In order to better understand this one may plot numerically the locus of the Hopf bifurcation point H^+ in the (r_R, K)-parameter plane, see Figure S8. Also included in this plot are the loci of Z_{21} ($r_R = d_R/K$), and Z_{23} ($r_R = d_R\rho_c$). These three loci, together with the line $K = 1/\rho_c$, form the four boundaries of the regions depicted in Figure S10 (Figure 1 in

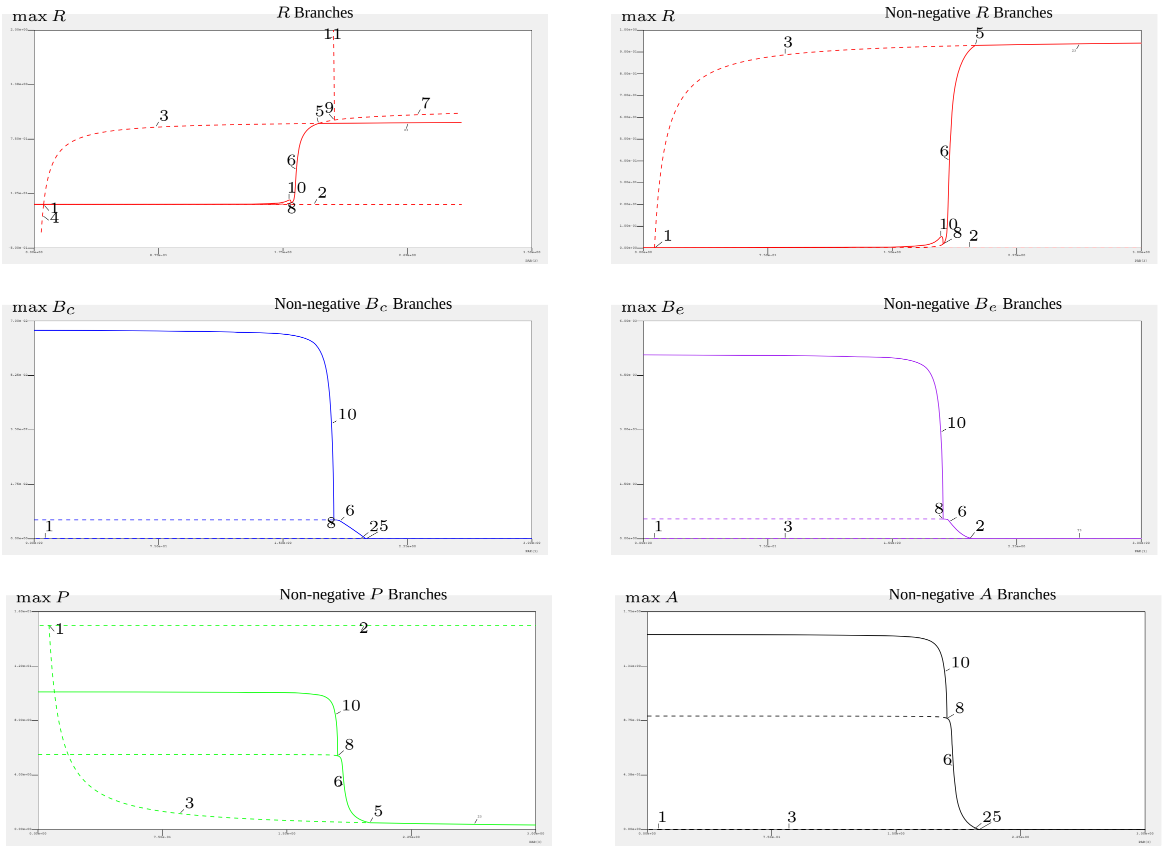

Figure S6: Bifurcation diagrams for Case 1 with $K = 15$. Diagram labels: (1) Z_{21}; (2) Σ_1; (3) Σ_2^+; (4) Σ_2^-; (5) Z_{23}; (6) Σ_3^+; (7) Σ_3^-; (8) H^+; (9) H^-; (10) Π^+; (11) Π^-.

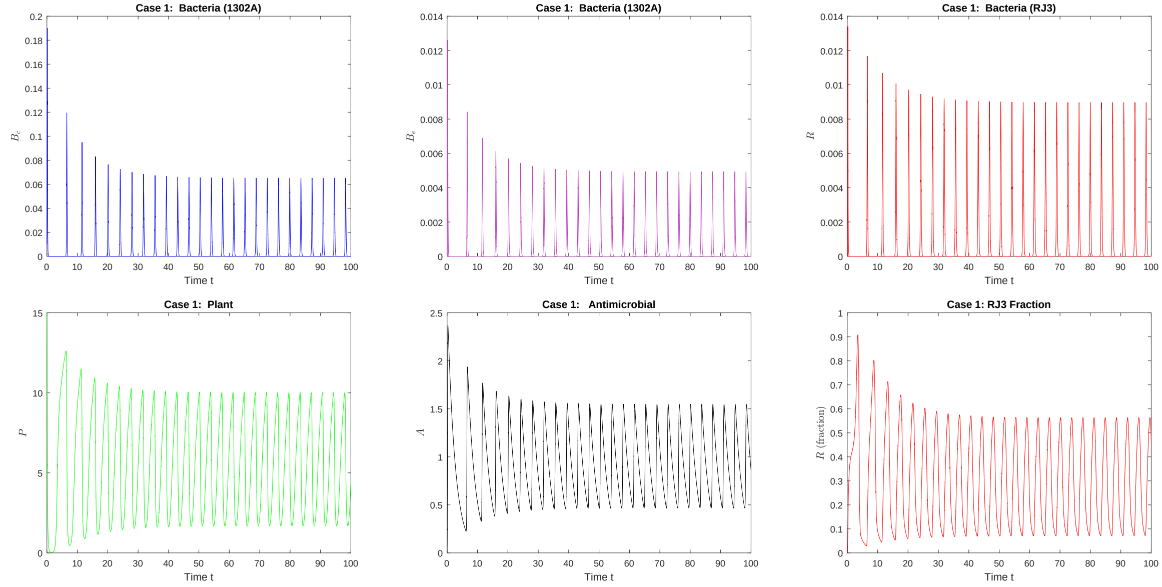

Figure S7: $K = 15$ and $r_R = 0.7$. Time plots illustrate convergence to a time-periodic co-existence limit cycle. Biologically this corresponds to the cyclic maintenance of the GI in the bacterial population within the plant host over time.

the main document). The locus of H^+ intersects the vertical K-axis at about $K = K^* = 11.2$. Asymptotically it also appears that $K \to \infty$ as $r_R \uparrow d_R\rho_c$. Consequently, Hopf bifurcations occur if and only if $K > K^*$. We summarize these results for Case 1 as follows:

- (large K) for $K > K^*$ there exist successive stable bifurcations $\mathbf{x}_2^* \to \mathbf{x}_3^* \to \mathbf{c}^+$ as r_R decreases;
- ('intermediate' K) for $1/\rho_c < K < K^*$ no Hopf bifurcation loci exist and the only stable bifurcation is from $\mathbf{x}_2^* \to \mathbf{x}_3^*$ as r_R decreases.

4.1.4 Fractional GI Loss

One may represent the loss of the GI in the bacterial population by plotting the eventual (i.e. as $t \to \infty$) fraction of RJ3 cells as a function of r_R. This is possible since for each value of r_R there appears to be a unique stable state, which is either a steady-state ($\mathbf{x}_2^*$ or $\mathbf{x}_3^*$) or a limit cycle ($\mathbf{c}^+$). We compute numerically the value of $R/(B_c + B_e + R)$ for each r_R, where the maximum value of $R(t)$, $B_c(t)$ and $B_e(t)$ is taken over one periodic cycle in the case of $\mathbf{c}^+$. The two distinct cases considered above ($K = 1$ and $K = 15$) are shown in Figure S9 .

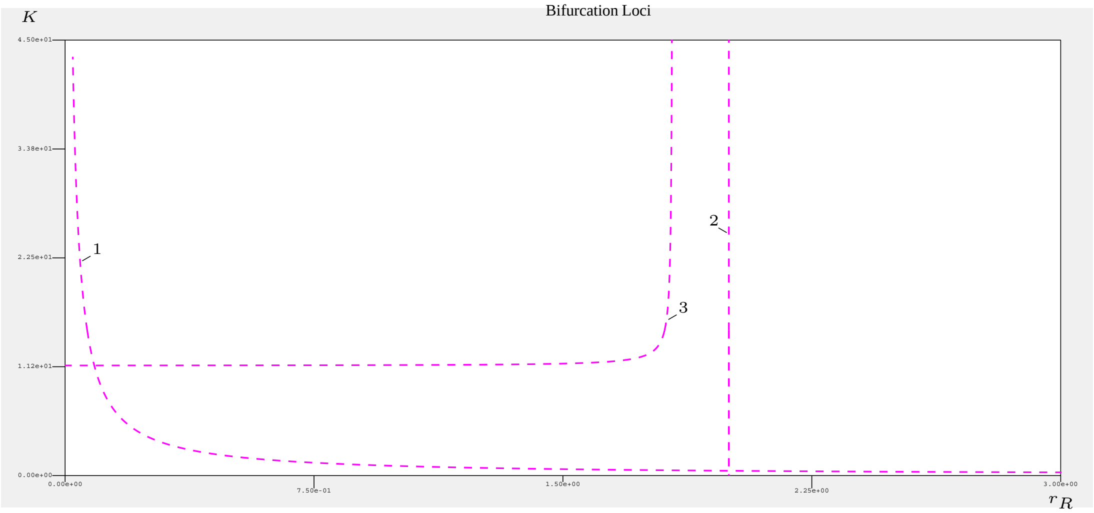

Figure S8: Case 1: Bifurcation loci in the (r_R, K)-plane. (1) Simple bifurcation locus Z_{21} ($r_R = d_R/K$) ; (2) Simple bifurcation locus Z_{23} ($r_R = d_R\rho_c$); (3) Hopf bifurcation locus H^+.

We remark that a value of r_R exists *slightly* smaller than $d_R\rho_c$ (or, equivalently, a value of ρ_R slightly smaller ρ_c) for which the RJ3 fraction is maintained at a stable value of 99.5%, i.e. for which 0.5% of the bacterial population still retain the GI - the situation we have observed experimentally (see Figures 4a-b of the main document).

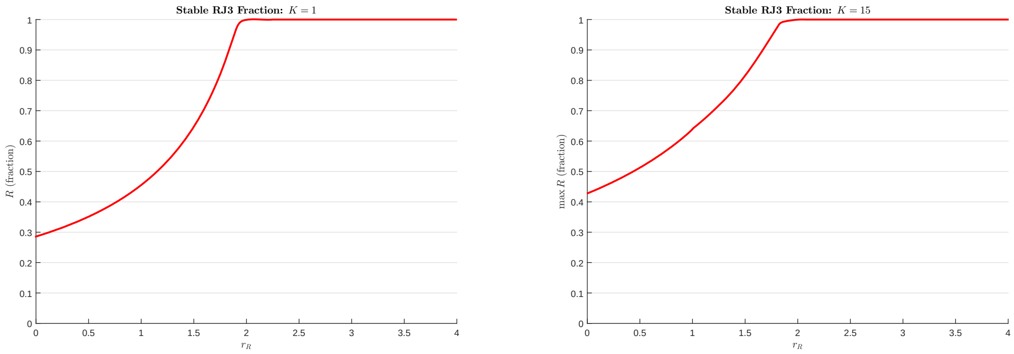

Figure S9: Case 1: Eventual RJ3 fraction for $K = 1$ (left) and $K = 15$ (right).

4.2 Case 2: $K < 1/\rho_c$

The situation here is much simpler. The bifurcation point Z_{23} corresponding to $r_R = d_R\rho_c$ is no longer bio-relevant since $R^* < 0$ when $r_R < d_R/K$. Consequently the Hopf loci $H^\pm$ are also no

longer bio-relevant. We summarize the bio-relevant results for Case 2 as follows:

- there exists a stable bifurcation $\mathbf{x}_2^* \to \mathbf{x}_1^*$ as r_R decreases

That is, $\mathbf{x}_1^*$ (plant cells only) is stable for $0 < \rho_R < 1/K$ and unstable for all $\rho_R > 1/K$ whilst $\mathbf{x}_2^*$ (plant and RJ3 cells only) is stable for all $\rho_R > 1/K$. Hence **GI retention is not possible**.

4.3 Summary

Here we summarise the results of our mathematical analysis and numerical simulations more conveniently and present the biological implications graphically. It will be expedient to introduce the notation

$$\hat{r} = d_R \rho_c. \tag{29}$$

Our results suggest the following characterisation:

- ⧫ (Small K) $0 < K < \hat{r}/d_R$.

  For $0 < r_R < d_R/K$, $\mathbf{x}_1^*$ (plant cells only, bacterial extinction) is the only stable state; for $\rho_R > d_R/K$, $\mathbf{x}_2^*$ (plant and RJ3 cells only) is the only stable state.

- ⧫ (Intermediate K) $\hat{r}/d_R < K < K^*$.

  For $0 < r_R < \hat{r}$, $\mathbf{x}_3^*$ (coexistence of 1302A and RJ3 bacterial cells) is the only stable state; for $r_R > \hat{r}$, $\mathbf{x}_2^*$ (plant and RJ3 cells only) is the only stable state.

- ⧫ (Large K) $K > K^*$.

  For $0 < r_R < r^+$, $\mathbf{c}^+$ (cyclic coexistence of 1302A and RJ3 bacterial cells) is the only stable state; for $r^+ < r_R < \hat{r}$, $\mathbf{x}_3^*$ (coexistence of 1302A and RJ3 bacterial cells) is the only stable state; for $r_R > \hat{r}$, $\mathbf{x}_2^*$ (plant and RJ3 cells only) is the only stable state.

Figure S10 portrays this characterisation in terms of genomic island loss or retention (Figure 1 of the main document).

5 Concluding Remarks

In these supplementary notes we have presented our mathematical model (Section 1) and performed a rigorous mathematical analysis for the existence, stability and local bifurcation of its steady-states (Sections 2 and 3). In Section 4 we presented the results of several numerical simulations performed for particular parameter values. For these chosen parameter values we obtained numerically the global structure and stability of the bifurcating branches and observed that Hopf

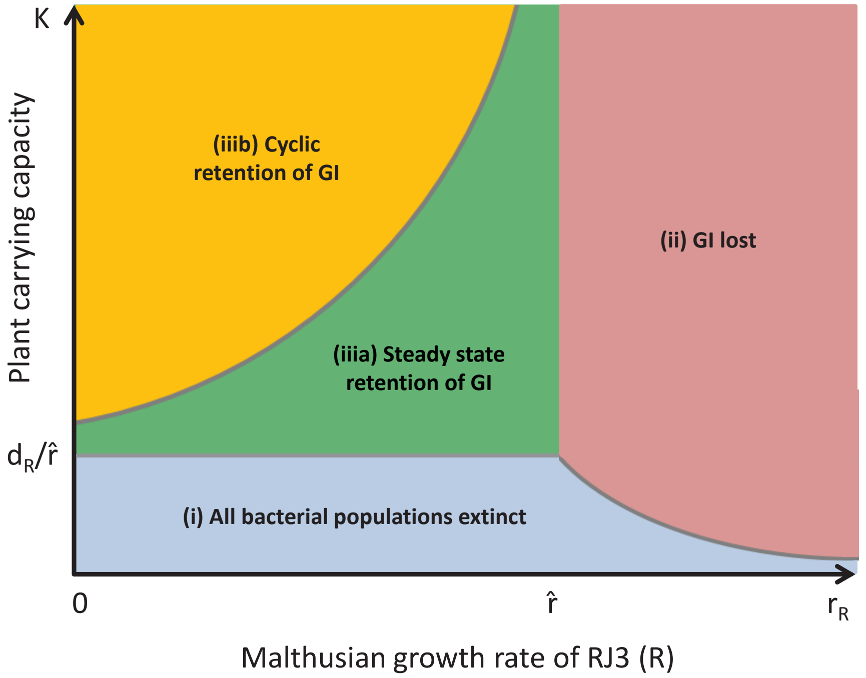

Figure S10: Characterisation of genomic island (GI) loss or retention in the (r_R, K)-parameter plane.

bifurcations exist when the carrying capacity K of the host plant is sufficiently large ($K > K^*$). In particular is was observed that the genomic island (GI) persisted in the population of bacterial cells (either at steady-state or time-periodically) if and only if K is sufficiently large ($K > 1/\rho_c$) and the reproductive ratio (ρ_R) of RJ3 cells is less than (ρ_c).

References

[1] N.F. Britton, *Essential Mathematical Biology*, Springer, (2003).

[2] M.G. Crandall and P.H. Rabinowitz, Bifurcation from simple eigenvalues, *J. Funct. Anal.* 8 (2), 321–340 (1971).

[3] M.G. Crandall and P.H. Rabinowitz, Bifurcation Perturbation of Simple Eigenvalues and Linearized Stability, *Arch. Rat. Mech. Anal.* 52 (2), 161–180 (1973).

[4] E.J. Doedel and B.E. Oldeman, *AUTO-07P: Continuation and Bifurcation Software for Ordinary Differential Equations*, Concordia University, Montreal, Quebec, Canada, 2009.

[5] G. Iooss and D.D. Joseph, *Elementary Stability and Bifurcation Theory*, Springer, (1997).

[6] Y. Kuznetsov, *Elements of Applied Bifurcation Theory*, Springer, (2004).
